# Comparative incidence of endophthalmitis after intravitreal dexamethasone implant versus anti-VEGF injections: a retrospective study

**DOI:** 10.1186/s40942-026-00824-3

**Published:** 2026-02-23

**Authors:** Iza D. Zabaneh, Tanner Dunn, Kortney Dunn, Patrick Williams

**Affiliations:** 1 Burnett School of Medicine, Texas Christian University, 1100 W Rosedale Street, Fort Worth, TX 76104 USA; 2 University of North Texas Health Science Center, Fort Worth, TX USA; 3 Texas A&M University Naresh K. Vashisht College of Medicine, College Station, TX USA; 4 Texas Retina Associates, Fort Worth, TX USA; 5 University of Texas Southwestern Medical School, Dallas, TX USA

**Keywords:** Endophthalmitis, Intravitreal injections, Dexamethasone implant, Anti-VEGF therapy, Ophthalmic complications, Incidence, Visual outcomes, Retina

## Abstract

**Background:**

Endophthalmitis is a rare but vision-threatening complication of intravitreal injections. Intravitreal dexamethasone implants and anti–vascular endothelial growth factor (anti-VEGF) agents are widely used, yet comparative real-world endophthalmitis rates remain incompletely defined. This study aimed to compare the incidence of endophthalmitis following intravitreal dexamethasone implant injections with that following anti-VEGF injections administered in outpatient retina practices.

**Methods:**

This retrospective cohort study included all intravitreal injections performed between January 2019 and January 2024 at participating practices. Endophthalmitis events consistent with post-injection exogenous endophthalmitis occurring within two weeks of intravitreal injection were identified through clinical records. Chi-square testing was used to compare incidence rates between dexamethasone implant and anti-VEGF injections. Patient characteristics, visual acuity outcomes, and treatment approaches were assessed for confirmed endophthalmitis cases.

**Results:**

A total of 330,572 intravitreal injections were analyzed, including 318,618 anti-VEGF injections and 11,954 dexamethasone implant injections. Endophthalmitis occurred more frequently after dexamethasone implant injections (0.125%, 15/11,954; 1.25 per 1,000 injections) than after anti-VEGF injections (0.033%, 106/318,618; 0.33 per 1,000 injections). This difference was statistically significant (χ² = 23.45, *p* = 2.3 × 10⁻⁷). Among dexamethasone-associated cases, 46.7% recovered to baseline or better visual acuity by six months, while the remaining patients experienced persistent visual deficits. Management commonly included intravitreal antibiotics with or without adjunct corticosteroids.

**Conclusions:**

Intravitreal dexamethasone implant injections were associated with a significantly higher incidence of endophthalmitis compared with anti-VEGF injections in this large outpatient cohort. Although nearly half of affected patients regained baseline vision, many had lasting impairment. These findings highlight the need for careful risk–benefit assessment when selecting intravitreal dexamethasone implants and underscore the importance of further study into modifiable risk factors and prevention strategies. A key limitation to consider is that clinically diagnosed endophthalmitis may include sterile intraocular inflammatory reactions following dexamethasone implant injection.

## Background

Endophthalmitis is a serious intraocular infection that can result in irreversible vision loss if not promptly identified and treated. It has been associated with connective tissue disorders, vasculitis, intraocular foreign material, and a variety of bacterial and fungal pathogens [1–4]. In the context of intravitreal injections (IVI), reported endophthalmitis incidence ranges from 0.02 to 0.08% in recent studies [5–8]. Although rare, endophthalmitis remains the most devastating complication of intravitreal injection, with estimated rates approaching 0.3% in some cohorts [2, 3, 9].

IVIs allow high drug concentrations to be delivered directly to the vitreous cavity, facilitating effective treatment of retinal and choroidal diseases such as age-related macular degeneration, diabetic retinopathy, macular edema following retinal vein occlusion, and uveitis [10–15]. Commonly administered agents include: intravitreal (IVT) corticosteroids, such as the dexamethasone implant, and anti-vascular endothelial growth factor (anti-VEGF) agents [16]. Post-injection endophthalmitis has often been linked to contamination from the patient’s skin or conjunctival flora, with coagulase-negative staphylococci and streptococci species being the most frequently isolated organisms [3, 16].

Despite the widespread use of both corticosteroids and anti-VEGF agents, comparative data on post-injection endophthalmitis risk between these two medication classes remain limited. Existing studies often involve small sample sizes or combine different corticosteroid preparations, making direct comparison challenging. Given the larger needle gauge required for corticosteroid implants, their immunosuppressive effects, and potential implant-related inflammatory responses, corticosteroid IVIs may carry a higher risk of infection than anti-VEGF agents.

This retrospective study examines and compares the incidence of endophthalmitis following IVI with the dexamethasone implant (Ozurdex, Allergan) versus anti-VEGF agents, as administered by multiple vitreoretinal specialists across U.S. outpatient centers over a 5-year period.

## Methods

### Study design and setting

This retrospective study evaluated the incidence of endophthalmitis following IVT injections of dexamethasone implant and anti-VEGF agents administered by vitreoretinal specialists across multiple outpatient centers within a single large retina practice from January 2019 to January 2024. Injection encounters were identified using Current Procedural Terminology (CPT) and HCPCS Level II codes corresponding to each agent (J0178, J9035, J2778, J7777, Q5128, J7312, and J0179). These structured identifiers allowed accurate categorization of all intravitreal injection types and agents administered during the study period. The exact number of participating centers and physicians could not be retrieved from the electronic dataset; however, all injections were performed by fellowship-trained retina specialists.

The primary objective of this study was to compare the incidence of post-injection endophthalmitis between intravitreal dexamethasone implant injections and anti-VEGF injections in a large outpatient retina practice cohort. Secondary objectives included evaluation of visual acuity outcomes following endophthalmitis and characterization of treatment approaches used in affected patients.

### Participants and data collection

The primary outcome measured was the incidence of endophthalmitis within two weeks post IVI. A two-week post-injection window was selected to capture the majority of clinically recognized injection-related endophthalmitis cases while minimizing inclusion of unrelated delayed intraocular infections. All intravitreal injections performed between January 2019 and January 2024 at participating outpatient retina centers were eligible for inclusion. Eligible cases included injections of both dexamethasone implants and anti-VEGF agents administered during the study period. Only clinically diagnosed cases consistent with post-injection exogenous endophthalmitis were included. Endophthalmitis was diagnosed based on clinical findings present with intraocular infection according to the physicians’ evaluation. Vitreous or aqueous tap with intravitreal antibiotic injection or pars plana vitrectomy for culture was performed in selected cases at physician discretion. Due to inconsistent availability and low sensitivity of office-based microbiologic testing, culture results were not required for case inclusion and were not used for outcome stratification. Cases of endophthalmitis flagged but determined to be unrelated to injections, such as endogenous endophthalmitis, were excluded from the total count of cases and final analysis. Data was extracted on visual acuity at three time points: baseline (defined as the visit immediately before injection), diagnosis, and six months after endophthalmitis, as well as any antibiotic treatments used.

### Injection protocols and procedures

All IVIs followed a structured protocol to minimize infection risk and there was some variability left to the discretion of the injecting physician. For IVT dexamethasone implant injections, a 22-gauge proprietary injector was used, while anti-VEGF injections employed 30–33-gauge needles with either pre-filled syringes, medicine drawn from vials, or, in the case of bevacizumab, syringes from a compounding pharmacy. Povidone-iodine was routinely applied for infection prophylaxis, consistent with standard infection-prevention guidelines. However, the electronic medical record did not uniformly record concentration and contact time.

Although procedural elements such as anesthetic technique (subconjunctival vs. topical lidocaine), use of lid speculum, sterile draping, conjunctival displacement, and needle entry approach varied by provider, all injections were performed by fellowship-trained vitreoretinal specialists following institutional infection control standards. The protocol prioritized preventing contamination from ocular flora, especially Staphylococcus epidermidis, and minimizing oral flora contamination through limited verbal communication and sterile technique. Topical antibiotics were not routinely used before or after injection. The absence of standardization and potential influence of heterogeneous injection techniques presented a study limitation due to the large number of physicians at multiple centers.

### Statistical analysis

Incidence rates of post-injection endophthalmitis were calculated separately for intravitreal dexamethasone implant injection and anti-VEGF injection groups. Group differences in incidence were assessed using chi-square testing. Relative risk (RR) with 95% confidence intervals (CI) was calculated to quantify comparative risk between groups. Statistical significance was defined as a two-tailed p value < 0.05.

Selection bias was minimized by including all eligible intravitreal injections within the defined study window identified using CPT and HCPCS billing codes. Due to the retrospective observational design and use of aggregate injection-level data without patient-level covariates, multivariable confounding adjustment and propensity score methods were not feasible. Potential confounders (e.g., injection indication mix, patient comorbidity burden, and provider technique heterogeneity) were considered qualitatively when interpreting results and are discussed as limitations. To account for the substantial imbalance in group sizes, outcomes were analyzed using incidence rates per injection and relative risk with confidence intervals rather than raw event counts.

## Results

### Incidence rates

**Fig. 1:**
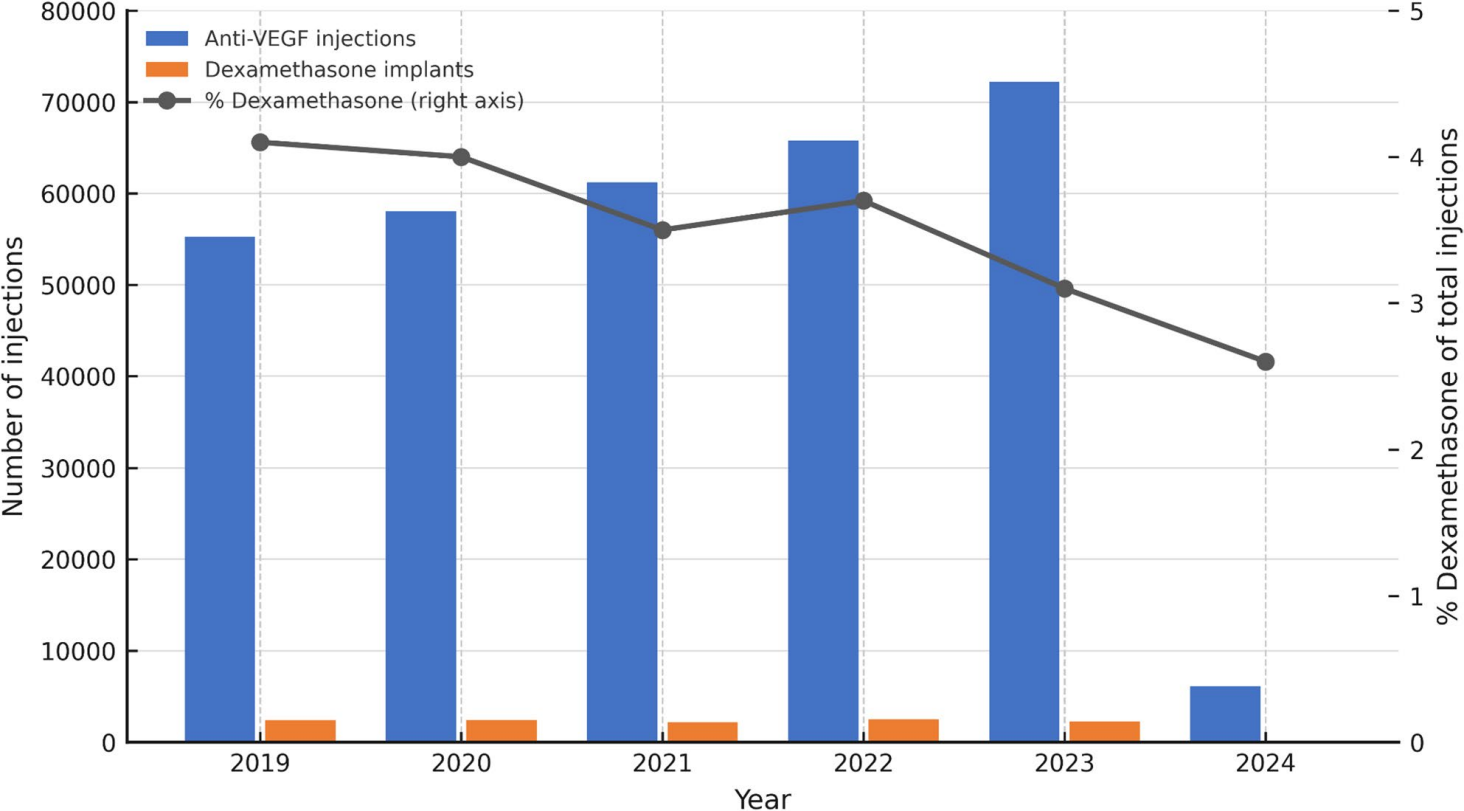
Shows total and annual distributions of intravitreal anti-VEGF and dexamethasone implant injections from 2019 to 2024. Bars represent yearly injection counts (left axis), and the line indicates the percentage of total injections accounted for by dexamethasone implants (right axis)

The study assessed the incidence of endophthalmitis following IVI of dexamethasone implant and anti-VEGF agents. Out of 11,954 dexamethasone implant injections, 15 cases of endophthalmitis were reported, resulting in an incidence rate of 0.125%, or 1.25 occurrences per 1,000 injections. In contrast, 318,618 anti-VEGF injections yielded 106 cases, with an incidence rate of 0.033%, or 0.33 occurrences per 1,000 injections. Chi-square analysis showed a statistically significant difference (χ² = 23.45, *p* = 2.29 × 10⁻⁷), indicating a higher risk of endophthalmitis following dexamethasone implant injections compared to anti-VEGF injections. Relative risk analysis demonstrated a significantly increased risk associated with dexamethasone implant injections (RR = 3.77; 95% CI: 2.20–6.47). The total and yearly distributions of anti-VEGF and dexamethasone implant injections between 2019 and 2024 are summarized in Fig. 1, which illustrates both cumulative injection totals and annual trends for each agent throughout the study period.

### Patient characteristics and clinical course

Patients who developed endophthalmitis following dexamethasone implant injections were analyzed based on their original diagnosis, intraocular pressure (IOP) at the time of diagnosis, and visual acuity at three time points: baseline (defined as the visit immediately before injection), at diagnosis, and six months after the diagnosis and treatment of endophthalmitis. Seven diagnoses warranting the use of dexamethasone implant were flagged in this study. These included: branch retinal vein occlusion (BRVO) with macular edema, central retinal vein occlusion (CRVO) with macular edema, macular edema, panuveitis, pars planitis, retinal edema, and type 2 diabetes mellitus (T2DM) with macular edema. Summarized in Table 1 are the clinical characteristics of patients who developed endophthalmitis following dexamethasone implant injections.

**Table 1:** Intravitreal Dexamethasone Implant Injection-Related Endophthalmitis: Patient Profiles and Visual Acuity Outcomes

Case #	Original Diagnosis	IOP at Diagnosis (mmHg)	Baseline VA (logMAR, Snellen)	VA at Diagnosis (logMAR, Snellen)	VA at 6 Months (logMAR, Snellen)
1	BRVO with macular edema	8	0.10, 20/25	1.85, 20/1420	0.48, 20/60
2	CRVO with macular edema	18	1.69, 20/980	2.10, 20/2520	1.83, 20/1350
3	CRVO with macular edema	16	1.60, 20/800	2.20, 20/3200	1.70, 20/1000
4	CRVO with macular edema	19	1.55, 20/710	2.00, 20/2000	1.65, 20/900
5	CRVO with macular edema	19	1.65, 20/890	2.10, 20/2520	1.75, 20/1120
6	Macular edema	25	0.88, 20/150	2.10, 20/2520	2.00, 20/2000
7	Panuveitis	10	0.18, 20/30	2.30, 20/4000	0.18, 20/30
8	Pars planitis	6	1.95, 20/1780	2.23, 20/3400	1.95, 20/1780
9	Pars planitis	6	1.90, 20/1600	2.20, 20/3200	1.90, 20/1600
10	Retinal edema	16	0.32, 20/42	0.75, 20/112	0.32, 20/42
11	Retinal edema	16	0.30, 20/40	0.80, 20/125	0.30, 20/40
12	Retinal edema	15	0.34, 20/44	0.70, 20/100	0.34, 20/44
13	Retinal edema	17	0.32, 20/42	0.75, 20/112	0.32, 20/42
14	T2DM with macular edema	14	0.25, 20/35	2.10, 20/2520	0.50, 20/63
15	T2DM with macular edema	14	0.30, 20/40	2.00, 20/2000	0.45, 20/56

Lists all 15 individual cases of endophthalmitis following intravitreal dexamethasone implant injection. Original diagnoses include branch retinal vein occlusion (BRVO) with macular edema, central retinal vein occlusion (CRVO) with macular edema, macular edema, panuveitis, pars planitis, retinal edema, and type 2 diabetes mellitus (T2DM) with macular edema. For each case, intraocular pressure (IOP) at diagnosis and visual acuity (VA) are shown at baseline (defined as the visit immediately before the causative injection), at the time of diagnosis, and six months after diagnosis. VA is expressed in both logMAR and approximate Snellen equivalents. Snellen equivalents are provided for clinical interpretability. For extreme visual acuity values, logMAR measurements represent the primary outcome metric

### Visual acuity and treatment outcomes

**Fig. 2:**
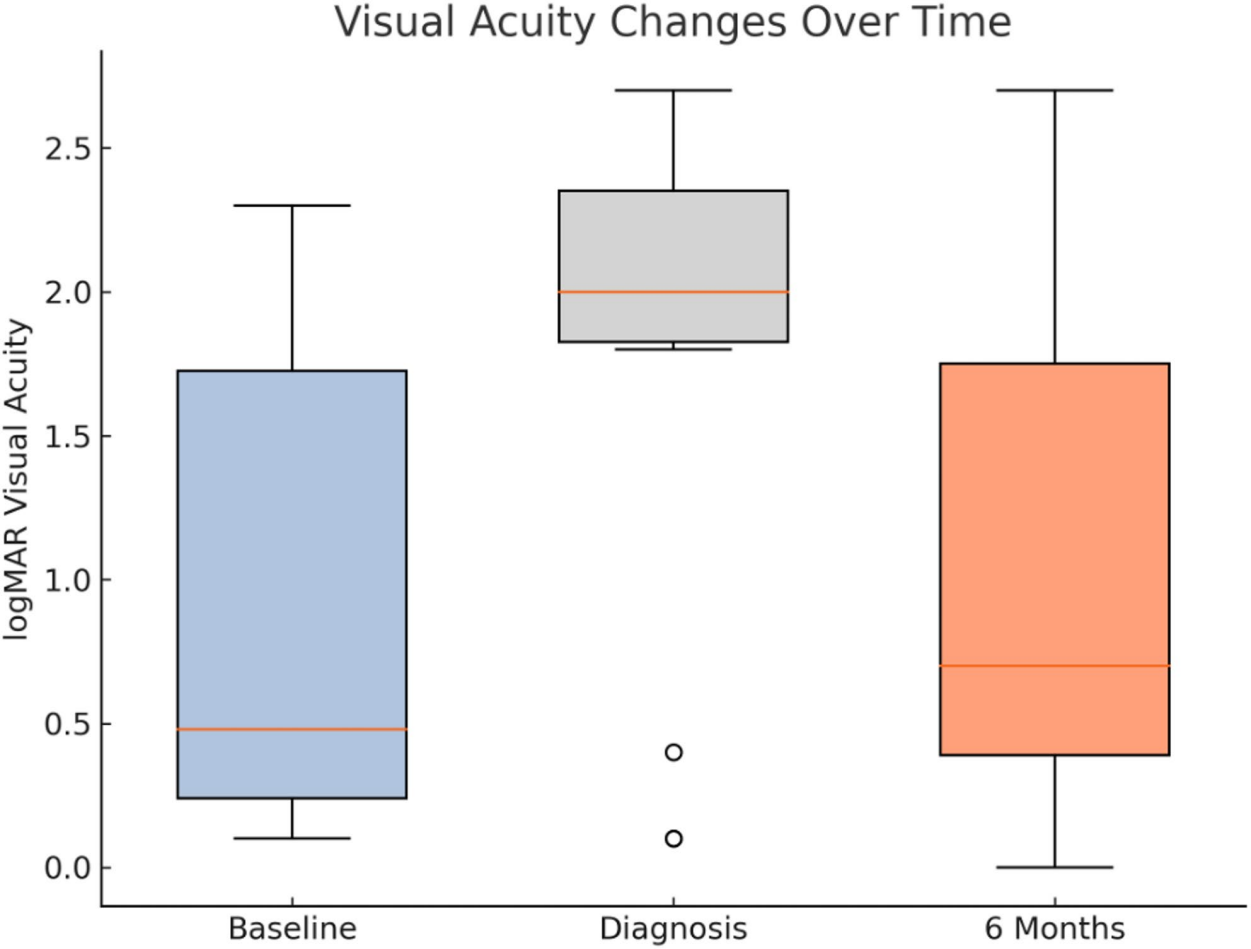
Visual acuity changes over time for 15 post-dexamethasone endophthalmitis cases, measured in logMAR. Depicts visual acuity changes over time for the 15 confirmed cases of endophthalmitis occurring after dexamethasone implant injection. Each case had at least six months of post-diagnosis follow-up. Baseline visual acuity was defined as the measurement obtained at a prior visit before the causative dexamethasone injection. Visual acuity is expressed in logMAR at baseline, at the time of endophthalmitis diagnosis, and at six months after diagnosis, illustrating variability in visual recovery following treatment

The clinical course of endophthalmitis cases varied based on the treatment approach. Interventions that were commonly administered for patients who suffered from endophthalmitis after dexamethasone implant injection included IVT antibiotics, such as ceftazidime and vancomycin, and adjunct corticosteroid therapy (e.g., prednisolone). Visual outcomes varied among patients, with approximately 46.67% of those affected returning to or exceeding their baseline visual acuity by the six-month mark. However, several cases experienced lasting visual acuity deficits, and over 50% did not return to baseline visual acuity. Baseline visual acuity was defined as the most recent measurement obtained at the clinic visit immediately preceding the causative dexamethasone implant injection. Visual acuity changes for the 15 cases of dexamethasone-related endophthalmitis are provided in Fig. 2, showing baseline visual acuity, acuity at diagnosis, and six-month follow-up acuity.

### Agent-specific incidences

**Fig. 3:**
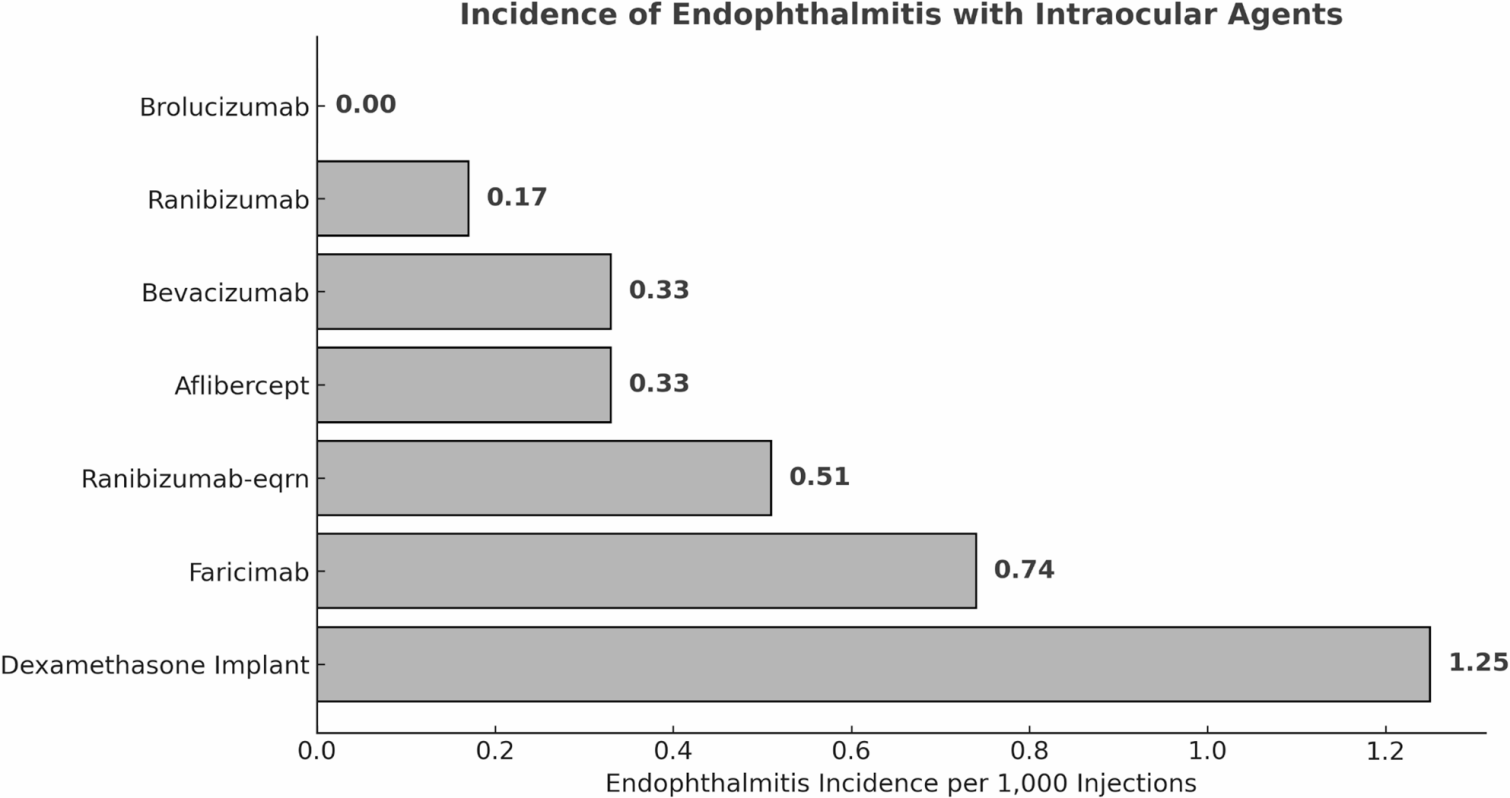
Incidence of Endophthalmitis following intraocular injections of various agents, measured per 1,000 injections. Depicts the incidence of endophthalmitis following intravitreal injections of various agents (anti-VEGF and dexamethasone) measured per 1,000 injections. This figure highlights the variability in endophthalmitis incidence across different injection agents used in the study

A detailed analysis of anti-VEGF agents revealed variability in endophthalmitis incidence rates. Aflibercept evidenced 58 cases of endophthalmitis out of 173,622 injections (0.0334% of total injections); ranibizumab had 7 cases out of 41,936 injections (0.0095% of total injections); bevacizumab had 26 cases out of 79,426 injections (0.0327% of total injections). Faricimab and brolucizumab demonstrated variable incidences, with no cases reported for brolucizumab. The incidence of endophthalmitis per 1,000 injections for each agent analyzed in this study is illustrated in Fig. 3. Faricimab and brolucizumab were introduced later in the study period and therefore contributed substantially fewer total injections compared with earlier-generation anti-VEGF agents, which limits the precision of agent-specific incidence estimates for these newer therapies. Agent-specific anti-VEGF injection volumes, endophthalmitis case counts, and corresponding incidence rates are summarized in Table 2.

**Table 2:** Anti-VEGF Agent Injection Volumes and Endophthalmitis Incidence

Agent	Total Injections	Endophthalmitis Cases	Incidence per 1,000 injections
Aflibercept (Eylea)	173,622	58	0.33
Bevacizumab (Avastin)	79,426	26	0.33
Ranibizumab (Lucentis)	41,936	7	0.17
Ranibizumab biosimilar (Cimerli)	1,971	1	0.51
Faricimab (Vabysmo)	18,936	14	0.74
Brolucizumab (Beovu)	2,727	0	0.00
**All Anti-VEGF Agents**	**318**,**618**	**106**	**0.33**

Incidence rates are expressed per 1,000 intravitreal injections. Injection counts reflect total exposure volume across the study period. Faricimab and brolucizumab were introduced later during the study window and contributed fewer injections compared with earlier-generation anti-VEGF agents

## Discussion

### Comparison of endophthalmitis incidence

While prior studies have established an increased risk of endophthalmitis following intravitreal corticosteroid injections, this present study provides a focused comparison between intravitreal dexamethasone implants and anti-VEGF agents within a large multi-center practice. A U.S. study involving 406,380 injections reported a 0.13% incidence of endophthalmitis after intravitreal steroid use, corresponding to an odds ratio of 6.9 compared with anti-VEGF injections [2]. Similarly, a French national cohort, encompassing more than 3.5 million injections found higher risk with corticosteroids (0.067%) than with anti-VEGF agents (0.020%) [11].

Meta-analyses have confirmed that anti-VEGF injections alone carry low endophthalmitis rates, approximately 0.05% per injection [5]. However, few large studies have directly compared dexamethasone implants with anti-VEGF injections until recently.

### Comparison with prior dexamethasone implant studies

Our results align with prior reports demonstrating that endophthalmitis following dexamethasone intravitreal implant is rare but slightly more frequent than after anti-VEGF injections. Stem et al. analyzed 3,593 dexamethasone implant injections and reported four cases of endophthalmitis, two being culture-proven after monoinjections (0.06% risk per injection) [17]. Rajesh et al. analyzed 6,015 injections and observed an incidence of 0.07%, further confirming the low overall risk [18]. Pancholy et al. compared 4,973 dexamethasone implant injections with more than 180,000 ranibizumab injections and found suspected endophthalmitis rates of 0.10% for dexamethasone versus 0.03% for ranibizumab, a statistically significant difference (*P* = .008), though culture-positive rates were similar across agents [19]. Samuelson et al. identified four cases of endophthalmitis among 3,925 dexamethasone implant injections (0.10%), with three culture-positive for gram-positive bacteria [20]. Kiristioglu et al. reviewed 3,430 dexamethasone implant injections and reported a 1.0% rate of sight-threatening, non-pharmacologic complications including cases of endophthalmitis [21]. Finally, Dhoot et al. analyzed more than 4 million intravitreal injections from the Vestrum Health Database and found suspected endophthalmitis rates of 0.107% for dexamethasone implants and 0.147% for triamcinolone. This is compared to 0.02–0.05% for anti-VEGF agents, confirming a higher relative risk associated with corticosteroid injections [22].

Taken together, these findings reinforce our observation that dexamethasone implants are associated with a slightly higher risk of post-injection endophthalmitis relative to anti-VEGF agents, yet the absolute incidence remains exceedingly low and consistent across studies.

### Clinical implications for treatment selection

IVI of therapeutic drugs has become one of the most common procedures in the field of ophthalmology during the past decade. At least 6 million IVIs have been carried out yearly since 2016 [23]. An estimated 8 million injections of IVIs, mostly anti-VEGF, corticosteroid, and complement inhibitors, were administered in 2023, and that number is expected to increase to 10 million by 2025 [24]. The anti-VEGF medications implicated in this study are considered first-line treatment options for retinal conditions such as diabetic macular edema, neovascular age-related macular degeneration, and retinal vein occlusion-related macular edema. With an excellent safety profile backed by a low incidence of serious complications, they are effective in improving patients’ visual acuity [25].

### Potential mechanisms for higher dexamethasone implant injection risk

Although endophthalmitis occurrences following dexamethasone implants is exceedingly rare [1, 2], research has highlighted that IVT corticosteroid use is linked to a significantly elevated risk—approximately sevenfold—compared to anti-VEGF injections [3]. This increased risk is partly attributable to the procedural and mechanical differences between the two techniques. The standard anti-VEGF injection employs a 30- or 32-gauge needle, whereas corticosteroid preparations use considerably larger needles: 27- or 25-gauge for triamcinolone and approximately 22-gauge for the dexamethasone implant. Moreover, anti-VEGF administration delivers only a small volume of fluid into the vitreous cavity, while corticosteroid injections introduce a solid implant that remains in situ, potentially causing a more sustained alteration of the vitreous environment. The larger needle size may create a more substantial wound tract, facilitating bacterial entry into the vitreous cavity.

In addition, the immunosuppressive effects of steroids are thought to contribute to heightened risk for endophthalmitis by lowering the threshold for bacterial inoculation. One study utilized rabbit eyes to compare the threshold for induction of endophthalmitis at various bacterial loads. It was discovered that eyes that received IVI of steroids required a significantly lower bacterial load when compared to non-steroid groups [4–6]. Another study suggests that corticosteroids act directly on local macrophages, impairing their phagocytic ability and rendering the patient more susceptible to pathogenic infection [7, 8]. Immunosuppressive conditions such as diabetes mellitus are also thought to increase risk of post IVI endophthalmitis [9].

Other studies indicate that corticosteroids may provoke sterile inflammation. This reaction can involve the release of pro-inflammatory cytokines, such as interleukin-6 (IL-6) and interleukin-8 (IL-8), in response to dexamethasone implant exposure [10]. This inflammatory response could imitate or worsen symptoms similar to infectious endophthalmitis, complicating clinical assessment and emphasizing the importance of close monitoring after injections.

### Preventative and management considerations

Prior literature consistently identifies coagulase-negative Staphylococcus and Streptococcus species, often derived from periocular or oral flora, as the most frequent causative organisms in post-injection endophthalmitis [16]. Effective management involves prompt intravitreal broad-spectrum antibiotics such as vancomycin and ceftazidime, with vitrectomy reserved for severe or non-responsive cases [7]. Preventive measures remain paramount: povidone-iodine antisepsis is the most evidence-based intervention to reduce bacterial load, while adjunctive strategies such as minimizing verbal communication and maintaining strict hand and eyelid hygiene further mitigate risk [26–29]. Routine use of prophylactic topical antibiotics is not recommended, as studies show no reduction in infection rates and potential promotion of resistant organisms [29, 30]. Collectively, adherence to these standard protocols remains the most effective means of minimizing endophthalmitis risk across all intravitreal injection types, including dexamethasone implants.

### Study strengths

Strengths of this study include its large real-world sample size (over 330,000 intravitreal injections) and inclusion of contemporary intravitreal agents across a multi-center outpatient retina practice setting. The five-year study period and consistent post-injection outcome window improve the reliability of incidence estimates and enhance generalizability to routine clinical practice.

### Study limitations

For this study, we were unable to distinguish between infectious endophthalmitis and sterile intraocular inflammation using the electronic medical records data. Case identification was based solely on clinical diagnosis rather than microbiologic confirmation, as the database used lacked data for laboratory cultures. This is a limitation for dexamethasone implant injections, which are known to appear clinically similar to sterile inflammatory reactions leading to possible overestimation of true infectious endophthalmitis rates. As a result, the absence of microbiological information, such as bacterial species and antibiotic sensitivities, made it impossible to analyze the specific pathogens involved and their associated risks.

Injection techniques and mitigations strategies also varied among providers in this multicenter study, with differences in needle size and aseptic methods. Minor procedural variations included differences in the type or concentration of povidone-iodine applied, the use of eyelid speculums, and post-procedure antibiotic drop practices. These variations could have affected infection rates and influenced the study’s overall findings.

Additionally, the study did not take into account important patient factors such as existing eye conditions, immune system suppression, or previous treatments, all of which might influence infection risk. The study also did not assess infection rates relative to the frequency of injections (e.g., 4-week versus 8-week intervals). Furthermore, patients receiving both anti-VEGF and corticosteroid injections at the same time were not specifically analyzed as a separate subgroup. In clinical practice, these injections are not typically performed during the same visit, so patients receiving combination therapy were not examined due to the assumption that infection risk will be independent.

A limitation of this retrospective analysis is the potential for residual confounding. Patients receiving dexamethasone implant injections may differ systematically from those receiving anti-VEGF therapy with respect to underlying diagnoses (e.g., uveitis versus age-related macular degeneration), as well as ocular risk factors such as prior vitrectomy, lens status, and ocular surface disease. Additionally, the study did not account for possible differences related to the use of compounded bevacizumab. Adjustment for these variables or subgroup analyses by treatment indication was not feasible because relevant covariates were inconsistently available across participating centers.

Another important limitation is that individual intravitreal injections were treated as independent events. Because many patients received multiple injections during the study period, outcomes may be subject to within-patient and provider-level clustering. Statistical approaches accounting for non-independence, such as generalized estimating equations or mixed-effects models, could not be implemented because the database did not reliably permit longitudinal linkage of injections at the patient or provider level across centers. Consequently, variance estimates may be underestimated and confidence intervals overly narrow.

## Conclusion

The incidence rates of endophthalmitis for IVT dexamethasone implants and anti-VEGF injections demonstrated a significant variation, with the former occurring at a rate of 1.25 per 1000 IVIs and the latter at 0.33 per 1000 IVIs. Though IVT injection-induced endophthalmitis is still rare within modern ophthalmology, the possibility of significant vision impairment or, worse, complete blindness renders it vital for ophthalmologists to carefully weigh the pros and cons of each treatment strategy. We believe that the larger needle gauge, inflammatory reactions linked to corticosteroid use, and immunosuppressive effects are the main causes of the elevated risk that is associated with IVT dexamethasone implants. Therefore, the findings of this study suggest a need for further research revolving around mechanisms of increased risk and safety protocols to mitigate complications such as endophthalmitis.

### Key clinical implications

This study evaluated real-world endophthalmitis incidence following intravitreal dexamethasone implant and anti-VEGF injections across a large outpatient retina cohort. The findings demonstrate a significantly higher relative risk of post-injection endophthalmitis associated with dexamethasone implants compared with anti-VEGF therapy, although absolute event rates remained low. Clinically, these results support careful risk–benefit assessment when selecting intravitreal corticosteroid implants and reinforce the importance of strict infection prevention practices to minimize rare but vision-threatening complications.

## Data Availability

The datasets used and/or analyzed during the current study are not publicly available due to patient privacy protections and institutional data use restrictions, but are available from the corresponding author on reasonable request.
